# Enrichment of colon cancer stem cells via polymeric porous filters with different zeta potentials

**DOI:** 10.1093/rb/rbag018

**Published:** 2026-02-09

**Authors:** Tzu-Cheng Sung, Ling-Chun Hung, Min Gao, Xuanyu Lin, Manman Yang, Xin Kang, Zeyu Tian, Ting Wang, Jian Gong, Jiandong Pan, Henry Hsin-Chung Lee, Akon Higuchi

**Affiliations:** State Key Laboratory of Eye Health, Eye Hospital, Wenzhou Medical University, Wenzhou, Zhejiang 325027, China; Department of Chemical and Materials Engineering, National Central University, Jhongli, Taoyuan 32001, Taiwan, China; State Key Laboratory of Eye Health, Eye Hospital, Wenzhou Medical University, Wenzhou, Zhejiang 325027, China; State Key Laboratory of Eye Health, Eye Hospital, Wenzhou Medical University, Wenzhou, Zhejiang 325027, China; State Key Laboratory of Eye Health, Eye Hospital, Wenzhou Medical University, Wenzhou, Zhejiang 325027, China; State Key Laboratory of Eye Health, Eye Hospital, Wenzhou Medical University, Wenzhou, Zhejiang 325027, China; State Key Laboratory of Eye Health, Eye Hospital, Wenzhou Medical University, Wenzhou, Zhejiang 325027, China; State Key Laboratory of Eye Health, Eye Hospital, Wenzhou Medical University, Wenzhou, Zhejiang 325027, China; Department of Clinical Laboratory, The Second Affiliated Hospital and Yuying Children’s Hospital of Wenzhou Medical University, Wenzhou, Zhejiang 325027, China; State Key Laboratory of Eye Health, Eye Hospital, Wenzhou Medical University, Wenzhou, Zhejiang 325027, China; Department of Surgery, Cathay General Hospital, Taipei 106, Taiwan, China; Graduate Institute of Translational and Interdisciplinary Medicine, National Central University, Taoyuan 32001, Taiwan, China; State Key Laboratory of Eye Health, Eye Hospital, Wenzhou Medical University, Wenzhou, Zhejiang 325027, China; Department of Chemical and Materials Engineering, National Central University, Jhongli, Taoyuan 32001, Taiwan, China; R&D Center for Membrane Technology, Chung Yuan Christian University, Taoyuan 320, Taiwan, China

**Keywords:** membrane filtration, cancer stem cell, cell sorting, colon cancer, positively charged filter

## Abstract

Colorectal cancer is one of the most prevalent malignant tumors worldwide, and cancer-initiating (CI) cells or cancer stem (CS) cells are critical for tumor progression. We purified CI/CS cells from colon cancer cells utilizing a membrane filtration method. We developed membrane filters with different surface charges (zeta potentials) by blending a negatively ionized polymer [poly(vinyl alcohol-itaconic acid), PVI] or a positively ionized polymer [poly(L-lysine), PLL] into poly(lactide-co-glycolic acid) (PLG). Suspensions of HT-29 colon cancer cells and PAT-3 cells (primary colon cancer cells from a colon cancer patient in this research) were permeated through unmodified PLG filters and modified PLG filters blended with and without PVI or PLL. The cells in the filtration and recovering solutions and migrating cells from the filters after filtration were evaluated to identify the cells in each fraction and which filter enriched CI/CS cells. CI/CS cells were evaluated for (a) CD44 and CD133 (CI/CS cell markers) expression by flow cytometry and immunostaining *in vitro*. Cells were also (b) evaluated by a colony formation assay *in vitro*, (c) evaluated for carcinoembryonic antigen (CEA) production by enzyme-linked immunosorbent assay *in vitro* and (d) evaluated for a xenograft tumorigenicity test using NOD.CB17-Prkdcscid/NcrCrl (NOD-SCID) mice *in vivo*. The results revealed that the migration of colon cancer cells from positively charged PLG/PLL filters increased the number of CI/CS cells efficiently and killed 83% of NOD-SCID mice after transplantation, whereas the other fraction of cells did not kill NOD-SCID mice. The filtration method through PLG/PLL filters is effective for purifying CI/CS cells from colon cancer cells.

## Graphical Abstract

**Graphical Abstract:**
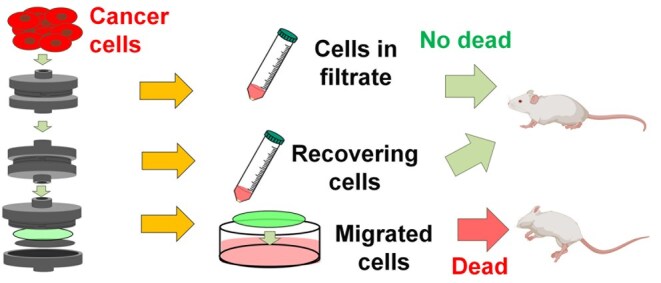

## Introduction

A small subset of tumor cells, which are known as cancer-initiating cells (CI cells) or cancer stem cells (CS cells), are considered to be responsible for tumor generation, growth, metastasis and recurrence. CI/CS cells hold the potential for self-renewal and differentiation into various types of cancer cells. The exact identification and specification of CI/CS cells from ocular, colon, lung, brain and other tumor cells is still challenging because of their rarity and the lack of specific, universally applicable markers [1–3]. Some surface markers have been investigated as colon CI/CS cell markers, such as Lgr5 [4–6], Musashi-1 [7, 8], aldehyde dehydrogenase 1 ALDH-1 [9–11], CD24 (cell adhesion molecule) [12, 13], CD44 (receptor for hyaluronan) [13–15], CD133 (prominin-1) [6, 16–21] and CD166 (activated leukocyte cell adhesion molecule, ALCAM) [22, 23]. CD133 may be the most important marker of CI/CS cells in colon cancer and other forms of cancer [6, 16–21]; however, hematopoietic stem cells also express CD133, and some research groups have reported a negative opinion regarding the usage of CD133 as a marker of colon CI/CS cells [24, 25]. Currently, no surface markers have been established well for colon CI/CS cells. Therefore, it is not meaningful to isolate colon CI/CS cells utilizing purification protocols, such as MACS (magnetic-activated cell sorting) or FACS (fluorescence-activated cell sorting) processes, which rely on surface markers of CI/CS cells for their purification mechanism. CI/CS cells are considered cells within tumors with the capability for tumorigenicity, proliferation and self-renewal when transplanted into animals [26]. However, many rat or mouse transplantation experiments are necessary to identify CI/CS cells. Therefore, colony formation analysis is typically performed instead of animal experiments to identify CI/CS cells, as this assay supports the assessment of the potential characteristics of CI/CS cells from cell lines for expansion *in vitro* [2, 27, 28].

Our research group created a filtration method for the purification of specific cells via specific filters [29–40]. In our previous studies, we used modified polyurethane porous (PUP) filters for the purification of hematopoietic stem cells from peripheral blood [33, 35, 36] and umbilical cord blood [32, 37, 38] using the filtration method. Adipose-derived stem cells (ADSCs) were also purified from human primary fat tissue solution using a nylon mesh (NM) filter [30, 31, 34, 39], PUP filter [30, 39, 40] and poly(lactide-co-glycolic acid)/silk screen (PLG/silk screen) filter [29, 30], where these filters had the pore size (*r*) of which ranged from 8 to 40 µm depending on the material chemistry and pore morphology.

In our previous study [41], CI/CS cells were purified from a colon cancer cell line (LoVo cells) utilizing NM filters (*r *= 20 or 11 μm) and PLG/silk screen filters (*r *= 21–32 μm). The LoVo cell solution flowed via the filters to yield a filtration solution. Then, the cell proliferation medium was passed via the filters to obtain the recovering solution where the cells attached to the filters were released into the recovering solution. Then, the PLG/silk screen filters were incubated with proliferation medium to release the migrating cells from the filters. The migrating cells from the filters displayed greater colony formation and displayed higher levels of CI/CS surface markers such as CD133 and CD44. Furthermore, compared with LoVo cells, the migrating cells secreted more carcinoembryonic antigen (CEA) protein and proliferated on conventional tissue cultivation plates (control experiments) [41]. However, the effect of the surface charge of PLG/silk screen filters on the purification of CI/CS cells was unknown in the previous study [41]. Furthermore, previous work did not proceed with direct evaluation of CI/CS cells *in vivo* by evaluation of tumor generation from transplantation of the cells into mice [41]. Therefore, we could not exactly evaluate CI/CS cell enrichment by the membrane filtration method via specific filters in the previous study [41]. Here, we prepared PLG/silk screen filters with different surface charges; one type was the previously developed PLG/silk screen filters (control experiments), and the other type was more positively ionized PLG/silk screen filters, which were prepared by adding (blending) a positively ionized polymer of poly-L-lysine (PLL) to yield PLG/PLL/silk screen (PLG/PLL) filters. We also prepared more negatively charged filters by adding (blending) negatively charged polymer [poly(vinyl alcohol-itaconic acid), PVI], yielding PLG/PVI/silk screen (PLG/PVI) filters. We evaluated which filters were preferable for isolating CI/CS cells from not only a colon cancer cell line (HT-29) but also human primary colon cancer PAT-3 cells, which were established from colon cancer tissue derived from a colon cancer patient in this study. Furthermore, we needed to confirm whether the colon cancer cells, which were purified through the membrane filtration method and showed high expression of CI/CS cell markers, were truly tumor-generating cells *in vivo*. Therefore, we verified that colon cancer cells with high CI/CS cell marker expression and colony formation intensity obtained using the membrane filtration method could easily generate tumors in immunodeficient mice by injecting the cells into the mice subcutaneously. The goal of this study is to investigate which surface charged filters are preferable to isolate CI/CS cells from primary colon cancer cells derived from patient colon cancer tissues, which would be useful to diagnosis the malignancy of patient colon cancer cells as well as to find an optimal drug treatment for colon cancer patients as a personalized treatment.

The membrane filtration method to purify CI/CS cells from cancer cells should be useful for other cancer cells, such as those derived from ocular melanoma, retinoblastoma, conjunctival squamous cell carcinoma and/or medulloepithelioma, in the future.

## Materials and methods

### Ethical statements

We received approval from the Ethics Committee of National Central University (NCU-109-010) for the animal experiments. Each animal experiment was performed in accordance with any applicable institutional or governmental guidelines or regulations. Isolation experiments using human colon tumor tissues from colon cancer patients were also approved by the ethics committee of Cathay General Hospital (IRB, CGH—P108082).

### Materials

HT-29 colon cancer cells (No. 60157) were received from the Food Industry Research and Development Institute, FIRDI (Taiwan, China). The chemicals, materials and biomolecules supplied in this work are listed in Supplementary Table S1. The other materials utilized in this work were obtained from Sigma–Aldrich (St. Louis, MO, USA).

### Preparation of PLG/silk screen filters

**Figure 1:**
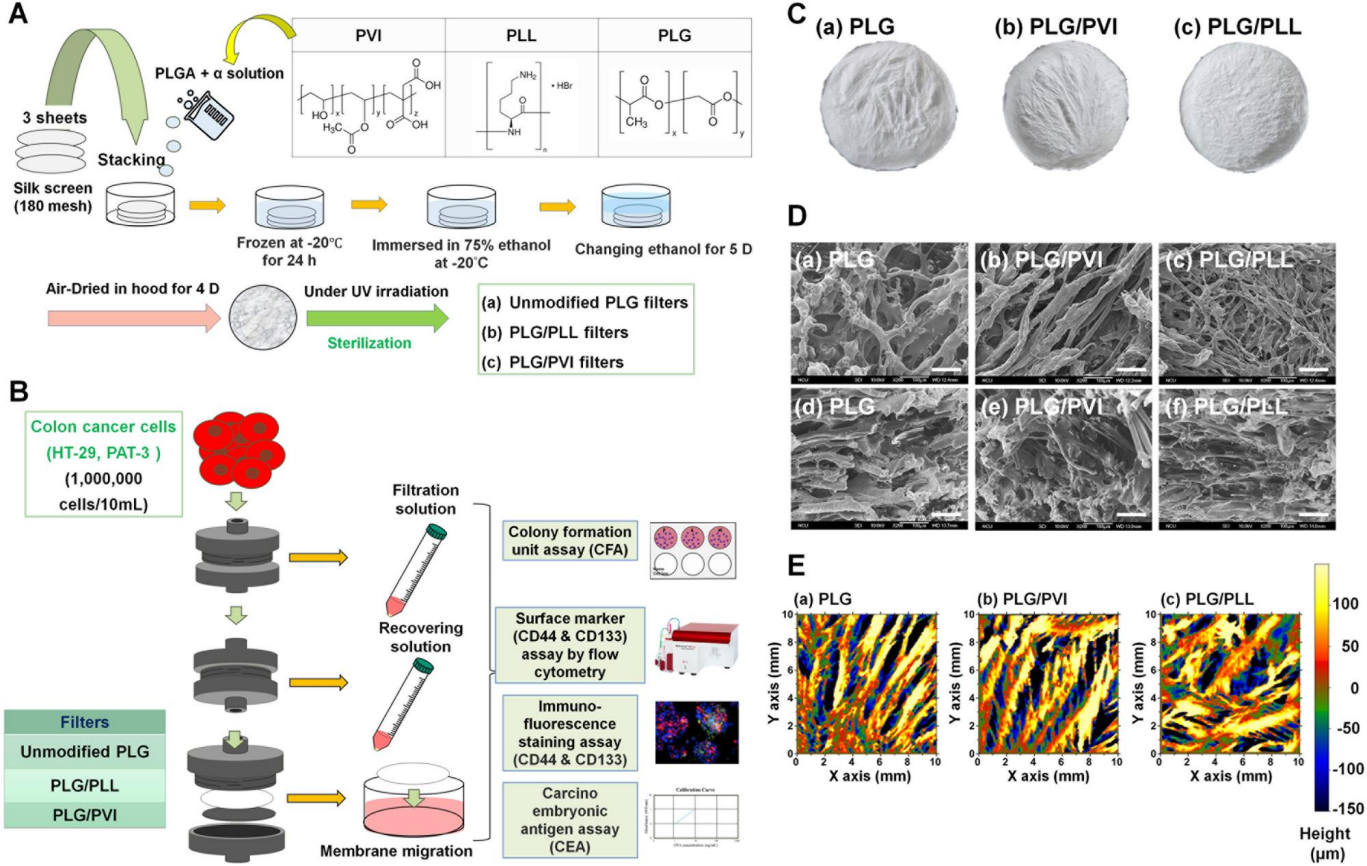
Experimental procedures. (**A**) Generation of PLG filters with or without modification with ionic polymers (PVI and PLL). Modified and unmodified PLG filters were prepared by a freezing–thawing extraction process. (**B**) Filtration and migration assays used to purify CI/CS cells from colon cancer cells. Colon cancer cell (HT-29 cells or primary colon PAT-3 cells) suspensions were permeated through filters (membranes) to obtain a filtration solution. Subsequently, the cell culture media were filtered to obtain the recovering solution. The filters were subsequently released from the filter holder and incubated in the cell culture media for 12 days to obtain migrating cells. The CI/CS cell surface marker expression of the cells was investigated by fluorescence microscopy or flow cytometry, colony-forming assays, CEA secretion determined by an enzyme-linked immunosorbent assay and a xenograft tumorigenicity assay by subcutaneous transplantation of cells into NOD/SCID mice. (**C**) Visual characterization of PLG filters (a), PLG filters blended with 0.5% PVI (PLG/PVI) (b) and PLG filters blended with 1% PLL (PLG/PLL) (c). (**D**) Morphology of PLG filters (a, d), PLG/PVI filters (b, e) and PLG/PLL filters (c, f) from the top visions (a-c) and cross-sectional visions (d-f) investigated by SEM. The bar represents 100 µm. (**E**) Surface roughness of PLG filters (a), PLG/PVI filters (b) and PLG/PLL filters (c) on the top surface, as analyzed utilizing a PRIMOS CR45 system.

A freezing-extraction method was used to prepare PLG/silk screen (PLG) filters [29, 42–46]. The filter preparation is schematically depicted in Figure 1A. PLG was dissolved in organic solvent (*N, N*-dimethylformamide, DMF) to make a 5 wt% PLG solution [47–52]. The PLG solution was warmed to 78–82°C for 90 min to ensure complete dissolution of the PLG particles. The hot PLG solution was gradually cooled to 25°C. Three sheets of silk screen mesh (180 mesh size, polyethylene terephthalate mesh) with circular dimensions (2.9 cm in diameter) were placed into glass Petri plates (3.0 cm in diameter), where grease was pre-coated to prevent the mesh from adhering to the glass surface. Three milliliters of the PLG/DMF solution were subsequently poured into glass Petri dishes, which contained three sheets of silk screen mesh, and frozen at −22°C for 2 days. The frozen PLG/DMF solid was inserted in 75% ethyl alcohol at −22°C for 4 days, and 75% ethyl alcohol was replaced by fresh 75% ethyl alcohol at −22°C three times a day to remove the solvent (DMF) from the PLG/DMF solid to form porous filters [PLG/silk screen (PLG) filters]. Then, the PLG filters were located in a fume hood at 20–25°C for 3 days and subsequently dried *in vacuo* at 32°C for 50 h to vaporize residual ethyl alcohol. Before the use of the PLG filters by the filtration method (Figure 1B), the filters were sterilized by UV exposure for 22–26 h.

### Preparation of modified PLG/silk screen filters

A positively charged polymer, PLL, was added to the PLG/silk screen filters (Figure 1A) to yield a more positive surface charge. First, 0.05 wt% PLL in a 5 wt% PLG solution was prepared. The same processes used to prepare the PLG/PLL silk screen (PLG/PLL) filters were subsequently performed, as described in the previous section.

A negatively charged polymer, poly(vinyl alcohol-co-itaconic acid)-L-lysine (PVI), was selected for addition to PLG filters (Figure 1A) to yield a more negative surface charge. PVI (0.025 wt%) was prepared in a 5 wt% PLG solution. The same processes used to prepare the PLG/PVI silk screen (PLG/PVI) filters were subsequently performed, as described in the previous section.

### Characterization of unmodified and modified PLG filters

The zeta potential (streaming potential) of the unmodified and modified PLG filters (2 cm × 1 cm) was evaluated in 0.01 mol/L KCl solution at pH = 7.0 and 25°C, which is adjusted by titration of 0.1 mol/L KOH solution utilizing a SurPASS 3 electrokinetic analyzer (Anton Paar, Graz, Austria).

The porous microstructures and pore sizes of the modified and unmodified PLG filters were evaluated using scanning electron microscopy (JEOL JSM-7000F, Tokyo, Japan). The pore size was evaluated using an ImageJ (https://imagej.nih.gov/ij/) system (NIH).

The roughness of the unmodified and modified PLG filters was evaluated utilizing a Primos-CR45 system (Camfield Scientific, Parsippany, NJ, USA).

### Colon cancer cell line cultivation

Colon cancer cells (HT-29 cells) were cultured in DMEM (Dulbecco’s modified Eagle’s media) containing 10% FBS (fetal bovine serum) at 37.0°C in a 5% CO_2_ incubator and passaged according to the typical protocol [53].

### Establishment of a primary colon cancer cell line from patient-derived colon tumor tissue

Primary colon cancer tissue was obtained from a colon cancer patient at Cathay General Hospital with ethics committee permission (IRB, CGH—P108082) where the patient agreed for us to isolate and use his/her colon cancer tissue for the research. The primary colon cancer tissue was stored in a centrifuge tube filled with saline solution after surgery. Once the primary colon cancer tissue was transported from the tube onto a plate on a clean bench, the digestion process was carried out within 45 min. The colon cancer tissue was dipped in a 12-well tissue culture polystyrene (TCP) dish with PBS supplemented with antibiotic-antimycotic (anti-anti) sequentially to wash and remove any impurities from the colon cancer tissue: (a) 4 washes with PBS supplemented with 5% anti-anti; (b) 4 washes with PBS supplemented with 2% anti-anti; (c) 2 washes with PBS supplemented with 1% anti-anti; and finally (d) 2 washes with PBS without anti-anti. Notably, 12 washes using anti-anti at different concentrations constitute the key process for establishing primary colon cancer cell lines with high probabilities, which we established from colon cancer experiments for more than 15 years. Following 12 washes, the tissue was transferred to a 10-cm TCP dish. The tissue was then fragmented into smaller pieces as much as possible (to approximately 1 × 1 × 1 mm^3^ in size). During this process, 2 mL of diluted collagenase type IV solution (100 U/mL) and 1 mL of CaCl_2_ solution (3 mM) were added to a 10-cm TCPS dish. The mixture was then transferred into a 50-mL tube using a 10-mL pipette and incubated in a 37°C water bath under continuous shaking using a laboratory shaking machine (NCS-1300, Eyela, Tokyo, Japan) for 1.5 h. During digestion, the larger tissue fragments were broken down into smaller pieces.

After the primary colon cancer tissue was digested, the enzymatic reaction was neutralized by adding an equal amount of fresh DMEM supplemented with 10% FBS to the tube. The solution was then filtered through a 100-μm pore-size cell strainer (130-098-463, Miltenyi Biotec, Bergisch Gladbach, Germany) to remove any undigested tissue fragments. The cells were subsequently centrifuged at 1500 rpm (377 *g*) for 7 min. After centrifugation, the supernatant was carefully removed, ensuring that the cell pellet at the bottom remained undisturbed. Then, 2.5 mL of ammonium-chloride-potassium (ACK) lysing buffer was added to the cells, which were subsequently incubated at 37°C for 6 min to lyse the red blood cells. Following the ACK treatment, an equal amount of DMEM supplemented with 10% FBS was added to neutralize the reaction, followed by centrifugation of the cell solution at 1500 rpm (377 *g*) for 7 min. The supernatant was removed, and PBS was added to wash the cells. The cell solution was then centrifuged again at 1500 rpm (377 *g*) for 8 min. Finally, the PBS was removed, and fresh DMEM supplemented with 10% FBS was added to resuspend the primary colon cancer cells. These primary colon cancer cells were then seeded onto Matrigel-coated TCP dishes. The successful adherence of primary colon cancer cells on the plates was evaluated under an inverted phase-contrast microscope after a few days of cell culture.

### Isolation of CI/CS cells by filtration and migration

Human colon cancer (HT-21) cell or primary colon cancer PAT-3 cell suspensions were filtered via unmodified or modified PLG filters using a batch-type filter module (C161, Millipore Corp.) (Figure 1B). The same filtration process as previously reported was performed in this study (Figure 1B) [29–31, 39, 40]. A total of 1 × 10^6^ cells in 10 mL of the colon cancer cell suspension were filtered at a constant temperature (25°C) and a filtration speed of 1.00 mL/min. The numbers of colon cancer cells in the filtration solution and the feed solution (*N*_p_ and *N*_f_, respectively) were estimated by flow cytometry (FS, BD Accuri™ C6, BD Biosciences). After the colon cancer cell suspension was filtered, the filter holder was placed upside down and the recovering solution consisting of cell proliferation medium (DMEM plus 10% FBS) was permeated through the filter holder, including the filters, at a filtration speed of 1.00 mL/min at 25°C. This process was necessary to release the cells attached to the filters into the recovering solution (Figure 1B). The total number of colon cancer cells in the recovering solution (*N*_r_) was also estimated utilizing FS.

The filtration rate and recovering rate were investigated from the number of cells in the filtration solution and recovering solution from Equations (1) and (2), respectively. The residual rate was calculated from Equation (3):


(1) $$\mathrm{Filtration}\;\mathrm{rate}\;\left(\%\right)=\left(N_{p}/N_{f}\right)\times 100\%,$$

(2) $$\mathrm{Recovering}\;\mathrm{rate}\;\left(\%\right)=\left(N_{r}/N_{f}\right)\times 100\%,$$

(3) Residual rate (%)=100 (%) – [Recovery rate (%)+Filtration rate (%)].

After the recovering solution (washing solution) was filtered, the filters were taken from the filter module and placed on cell proliferation plates containing culture medium (DMEM containing 10% FBS). The residual cells that attached to the filters started to migrate out from the filters onto the cell proliferation plates when the filters over the course of 12 days at 37°C in a 5% CO_2_ incubator. The total number of migrated cells was estimated utilizing FS.

The expression of CI/CS cell markers (CD44 and CD133) on the cells in the recovering solution, filtration solution and feed solutions as well as on the migrating cells was analyzed using FS after staining with 7-AAD.

### Colony formation assay

The colony formation assay using soft agarose is an analytical protocol to characterize the anchorage-independent proliferation abilities of cells *in vitro* [28] and is typically utilized for the identification of CI/CS cells [28, 54–58]. The experimental process was performed according to a previously reported method [28, 54] with some modifications, as follows: (1) A 3 wt% 2-hydroxyethyl agarose solution was prepared using 2-hydroxyethyl agarose with ultrapure water, after which the agarose solution was autoclaved and stored at 20–25°C until use. (2) A 3 wt% agarose solution was diluted with pure water at 46°C to obtain a 0.59 wt% agarose solution, and 2 mL of the 0.59 wt% agarose solution was injected into each well of 6-well dishes at 46°C. The 6-well dishes were placed on a flat surface in a refrigerator for 1 h to allow the agarose solution to become solid at 4°C, and the agarose gel was used for the colony formation assay. (3) The 0.59 wt% agarose solution was made by mixing 8 mL of warm DMEM with 10% FBS, and 2 mL of the 3% agarose solution was prepared. Then, the suspension of colon cancer cells was mixed with 0.59% agarose solution at a 1:1 ratio. Afterward, 1 mL of the mixed cell–agarose solution with 2 × 10^4^ cells was injected onto the agarose gel surface in the 6-well plates. The 6-well plates were located in a refrigerator for 16 min such that the cell–agarose mixed layer was solid at 4°C. After the cell–agarose mixture solidified, the cell–agarose plates were incubated at 37°C for 21 days; 100 μL of the cultivation media was added to the cell-agarose plates once every 3 days to avoid drying. The colony appearance could be analyzed and visualized on a microscope three weeks after cell seeding. The colonies with diameters ≥495 μm were considered colonies, and the number of colonies was calculated.

### CEA secretion analysis

The amount of CEA secreted by the colon cancer cells was evaluated utilizing EIA (enzyme-linked immunosorbent assay) kit [59] after the cells were filtered via unmodified and modified PLG filters. The cells in the filtration and recovering solutions and migrating cells were allowed to proliferate on TCP dishes for 7 days. Then, the cells were released with trypsin-EDTA solution and centrifuged at 395 × *g* for 7 min to obtain the cell pellets. The cell pellets were injected into a tube (2 mL) containing DMEM (198 μL). Then, we lysed the cells in the tube by freezing them in liquid N_2_ for 25 s and subsequently thawing them in a 36–38**°C** warm solution for 60 s. The freezing–thawing procedure was continuously performed four times. After centrifugation at 395 × *g* for 7 min, the concentration of the supernatant was investigated to determine the rate of CEA secretion per cell. The CEA secretion rate was estimated from the following formula:


(4) $$\mathrm{CEA}\;\mathrm{secretion}\;\mathrm{rate}\left(ng/10^{6}\mathrm{cells}\right)=C*V*10^{6}/Z,$$

where *C* is the concentration of CEA in the supernatant generated from colon cancer HT-29 or primary colon cancer PAT-3 cells, *Z* is the cell number and *V* is the medium volume (200 μL).

### A xenograft tumorigenicity assay

The presence and quantification of CI/CS cells were evaluated *in* *vivo* using a xenograft tumorigenicity assay following a standard procedure. Colon cancer cells before and after filtration through PLG/PLL filters were obtained by treating the cells with a cell dissociation solution, after which the cell pellets were suspended in DMEM/F12 medium with Matrigel after centrifugation. A total of 8.0 × 10^6^ cells per mouse were injected subcutaneously into NOD-SCID (nonobese diabetic/severe combined immunodeficiency, NOD.CB17-Prkdcscid/NcrCrl) mice (Beijing Vital River Laboratory Animal Technology, Beijing, China). Cells from the following conditions were injected into the mice: (a) PBS (control, no cells injected); (b) PAT-3 cells before filtration; (c) PAT-3 cells in filtration solution after filtration via PLG/PLL filters; (d) PAT-3 cells in recovering solution after filtration via PLG/PLL filters; and (e) PAT-3 cells migrating from PLL filters after filtration via PLG/PLL filters. The tumor size and condition (live or dead) of each mouse were evaluated once every week for 6 weeks.

### Statistical analysis

Each experiment was performed using four different samples under the same conditions. All data were expressed as mean ± standard deviation (SD) and analyzed using one-way analysis of variance (ANOVA) with *post* *hoc* tests (Bonferroni test) using GraphPad Prism 10.6.1.892 (GraphPad Software, Boston, MA, USA). Significance was set at *P *< 0.05, while *P *> 0.05 was considered to be statistically non-significant (n.s.).

## Results and discussion

### Characterization of the filters

We intended to purify CI/CS cells from colon cancer cells by filtration via unmodified PLG and modified PLG (PLG/PVI and PLG/PLL) filters with a wide range of surface charges (zeta potentials) in this study (Figure 1A and B). We prepared unmodified PLG filters and modified PLG/PVI and PLG/PLL filters because the PVI polymer has negatively charged sites (itaconic acid) and because PLL is a positively charged polymer (Figure 1A). PVI and PLL were selected as blended polymers into PLG porous membranes to control surface charges of the membranes because both PVI and PLL could be dissolved in DMF, which is a good solvent of PLG. An overview of the top surface morphologies of the filters, which are not-transplant but white, is shown in Figure 1C, indicating that these filters have porous structures. The front top surface and cross section of these filters were also examined by SEM (Figure 1D). Both the top surface and the cross section show porous morphologies for the unmodified PLG and modified PLG (PLG/PVI and PLG/PLL) filters. The average pore size of filters is among the most important parameters in filter characterization, and the pore size of the filters was evaluated from the top surface of the SEM images using ImageJ; the results are displayed in Table 1. The average pore sizes of the three different (PLG, PLG/PVI and PLG/PLL) filters ranged from 37.6 to 43.4 µm, and the size of colon cancer cells is typically around 10 µm. The porosities of the three filters were also found to be similar, ranging from 50.9 to 55.3%.

**Table 1:** Some characteristic properties of unmodified and modified PLG membranes with comparison to those of TCP dishes.

Membranes	PLG	PLG/PVI	PLG/PLL	TCP
Average pore size (µm)	53.4 ± 17.9	40.4 ± 16.2	37.6 ± 7.1	0
Porosity (%)	53.8 ± 1.9	55.3 ± 3.3	50.9 ± 5.9	0
RMSR (µm)	117.3 ± 57.6	76.7 ± 26.6	65.9 ± 8.8	10.1 ± 1.8
Zeta potential (mV)	−22.7 ± 0.9	−27.4 ± 0.9	17.9 ± 2.2	−19.9 ± 1.1

Surface roughness was also evaluated using a Primos CR45 system, and the results are displayed in Figure 1E. Relatively rough surfaces were observed for both the unmodified and modified PLG filters. The root mean square (RMS) roughness was evaluated using the Primos CR45 system, and the results are displayed in Table 1. Compared with the modified PLG filters, the PLG filters had relatively high RMS roughness, i.e. 117.3 µm, whereas the modified PLG/PVI and PLG/PLL filters had lower RMS roughness, i.e. 76.7 and 65.9 µm, respectively. The blending of ionic PVI and PLL polymers with PLG may result in filters with a smoother surface.

The zeta potential of three different (PLG, PLG/PVI and PLG/PLL) filters was evaluated from the streaming potential, and the results are displayed in Table 1. The zeta potential of the PLG/PVI filters was more negative (−27.4 mV) than that of the unmodified PLG filters (−22.7 mV), which originated from the negatively charged polymer PVI, whereas the zeta potential of the PLG/PLL filters was more positive (17.9 mV) than that of the unmodified PLG filters, which was attributed to the positively charged polymer PLL.

### Permeation of HT-29 colon cancer cells and PAT-3 primary colon cancer cells

**Figure 2:**
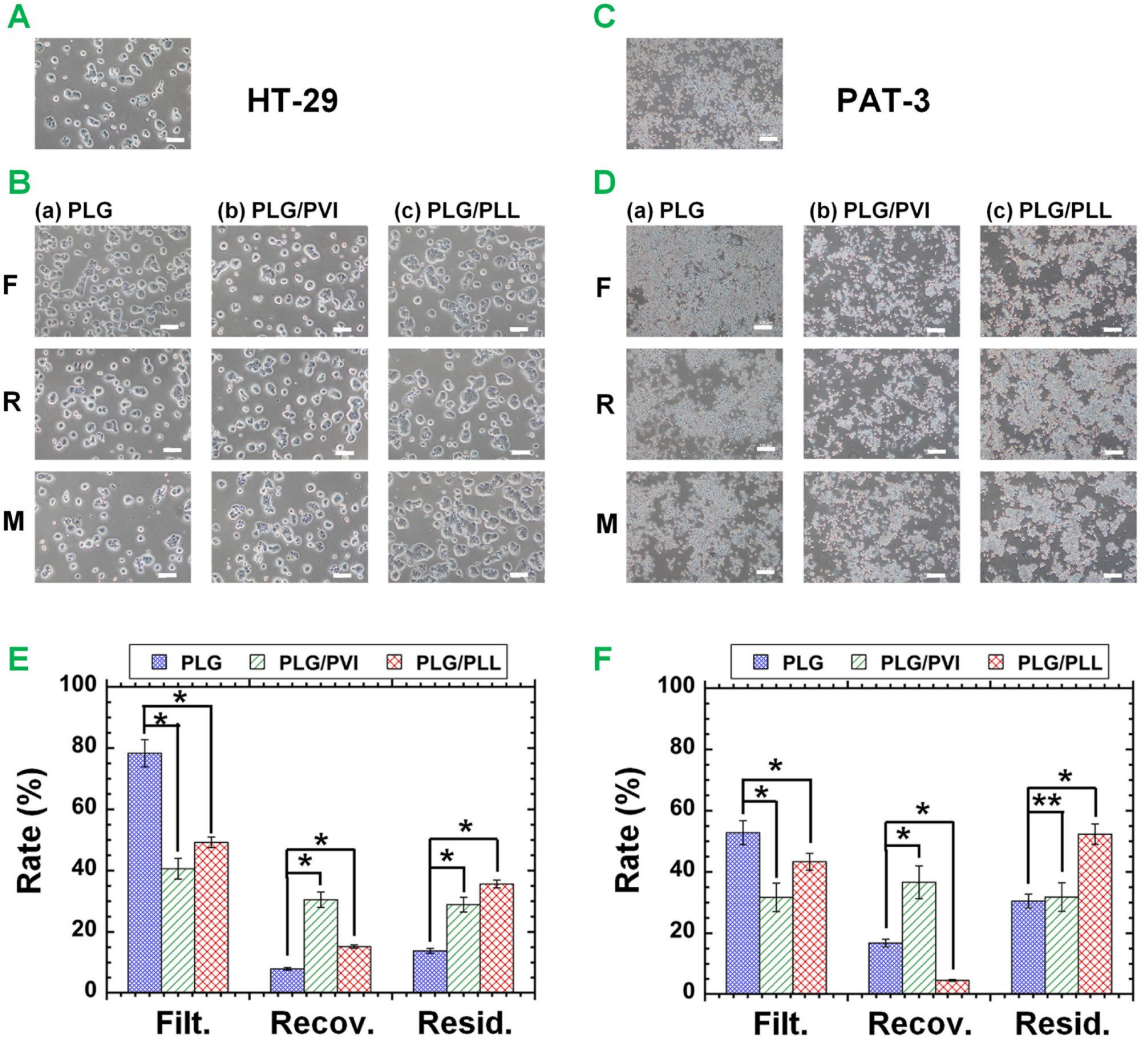
Filtration of colon cancer cells through unmodified and modified PLG filters. (**A**) HT-29 cell morphology on TCP dishes. (**B**) HT-29 cell morphology in filtration solution (F) and recovering solution (R) and on migration (M) from PLG filters (a), PLG/PVI filters (b) and PLG/PLL filters (c). the bar represents 100 µm. (**C**) PAT-3 cell morphologies on TCP plates. (**D**) PAT-3 cell morphology in filtration solution (F) and recovering solution (R) and on migration (M) from PLG filters (a), PLG/PVI filters (b) and PLG/PLL filters (c). the bar represents 100 µm. (**E**) Filtration rate (Filt.), recovering rate (Recov.) and residual rate (Resid.) of HT-29 cells through PLG, PLG/PVI and PLG/PLL filters. (**F**) Filtration rate, recovering rate and residual rate of PAT-3 cells through PLG, PLG/PVI and PLG/PLL filters. *P < 0.05; **P > 0.05.

HT-29 cells were selected as colon cancer cells and filtered through unmodified PLG and modified PLG (PLG/PVI and PLG/PLL) filters, and the cells in the filtration and recovering solutions as well as the migrating cells were evaluated under an inverted phase-contrast microscope; the cell morphologies are displayed in Figure 2A and B. The HT-29 cells before filtration (Figure 2A) and after filtration through three different filters (Figure 2B) showed the same cell morphology; we did not observe any differences in cell morphology between before and after permeation through the filters in this study. Furthermore, the cells in the filtration solution and recovering solution showed almost the same morphologies as the migrating cells in this study.

We also evaluated a different colon cancer cell line, which was established from a colon cancer patient in our laboratory (PAT-3 cells), for the filtration of primary colon cancer cells via filters, and their morphologies are displayed in Figure 2C and D. The morphology of the PAT-3 cells differed slightly from that of the HT-29 cells, with smaller cell aggregates but no large colonies, as observed in the HT-29 cells. However, the morphology of PAT-3 cells did not differ before and after filtration through three different filters (PLG, PLG/PVI and PLG/PLL) in this work. Furthermore, the PAT-3 cells in the filtration and recovering solutions showed almost the same morphology as the migrating cells, similar to the HT-29 cells.

The numbers of HT-29 cells and PAT-3 cells in the feed solution, filtration solution and recovering solution were evaluated by FS, thereby enabling the calculation of the permeate rate, recovering rate and residual rate from Equations (1)–(3). The results are shown in Figure 2E for HT-29 cells and Figure 2F for PAT-3 cells. The permeation rate through PLG filters was higher than that through PLG/PVI or PLG/PLL filters for both HT-29 cells and PAT-3 cells. The recovering rate through PLG/PVI filters was greater than that through other filters, such as PLG or PLG/PLL filters, for both HT-29 cells and PAT-3 cells. The residual rate through PLG/PLL filters was the highest among the residual rates through unmodified and modified PLG (PLG and PLG/PVI and PLG/PLL) filters. This is because the PLG/PLL filters have a positive zeta potential (Table 1) and can strongly interact with negatively charged cells electrostatically. Especially, CI/CS cells are expected to be highly adhesive and migrating cells because of high malignant characteristics. Therefore, CI/CS cells remained on the positively charged PLG/PLL filters and migrated out from the PLG/PLL filters as the migrated cells, when the filters were incubated in the culture medium.

### CI/CS cell marker expression in colon cancer cells before and after filtration

**Figure 3:**
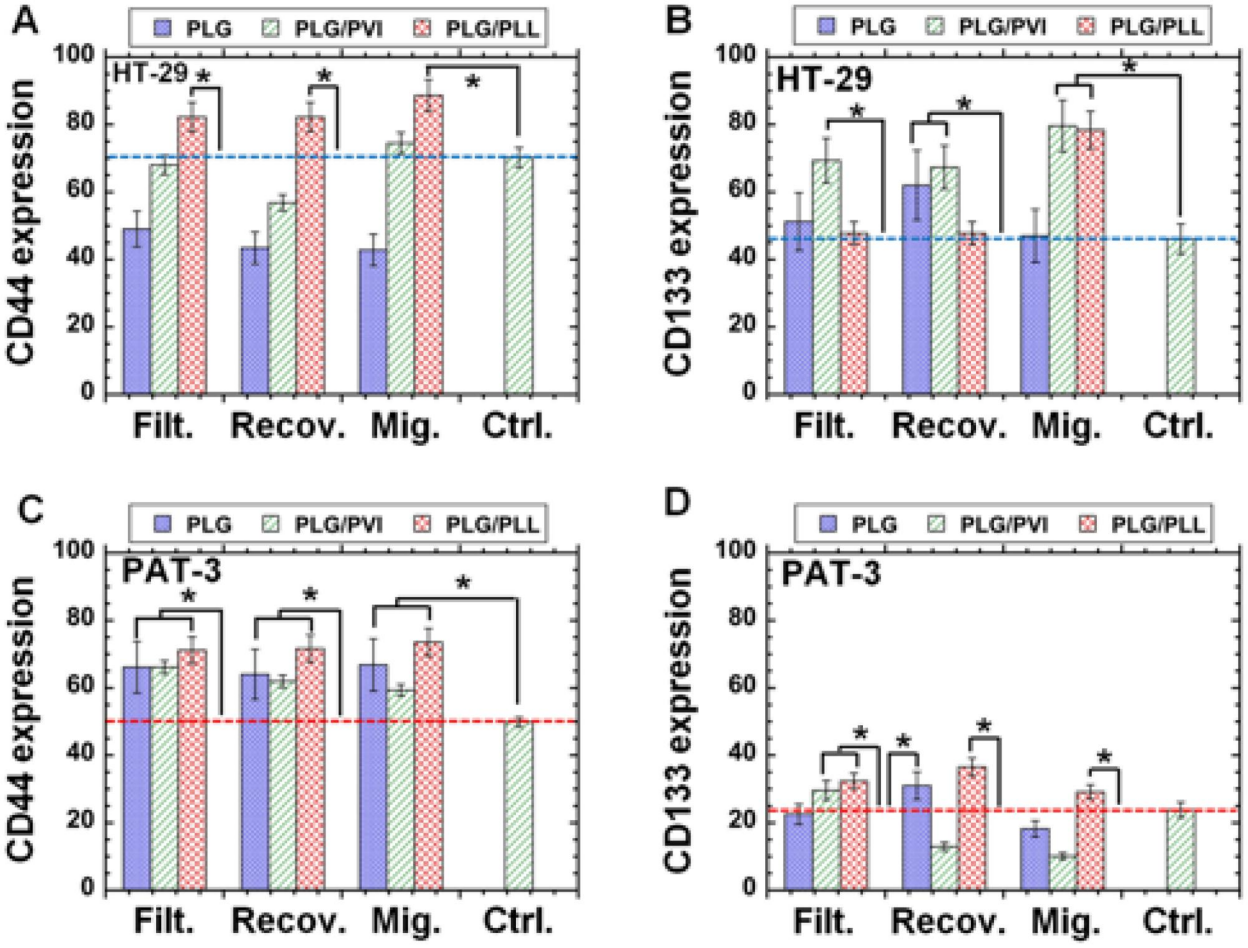
Expression of CI/CS cell markers by HT-29 and PAT-3 cells evaluated by FS after filtration via unmodified and modified PLG filters (membranes). (**A**, **B**) HT-29 cells expressing CD44 (**A**) and CD133 (**B**) in the filtration solution (Filt.) and recovering solution (Recov.) and on migration (Mig.) after filtration through PLG, PLG/PVI and PLG/PLL filters. (**C**, **D**) PAT-3 cells expressing CD44 (**C**) and CD133 (**D**) in the filtration solution and recovering solution and on migration after filtration through PLG, PLG/PVI and PLG/PLL filters. The dotted line displays CD44 (**A**, **C**) and CD133 (**B**, **D**) expression by unfiltered cells allowed to proliferate on TCP plates. *P < 0.05.

To investigate whether filtration through colon cancer cells enriched CI/CS cells, we evaluated CI/CS cell marker (CD44 and CD133) expression in colon cancer cells (HT-29 cells and primary PAT-3 cells) using FS before and after filtration through unmodified PLG filters and modified PLG (PLG/PVI and PLG/PLL) filters, and the results are summarized in Figure 3A and B for HT-29 cells and Figure 3C and D for PAT-3 cells.

In terms of CD44 expression on the cells (Figure 3A and C), the cells in the filtration and recovering solutions as well as the cells migrating through the PLG/PLL filter presented higher CD44 expression than the cells before filtration (control) for both HT-29 cells and PAT-3 cells. The cells migrating through the PLG/PVI filters showed greater CD44 expression than the cells before filtration for both HT-29 cells (Figure 3A) and PAT-3 cells (Figure 3C). Neither HT-29 cells nor PAT-3 cells in filtration and recovering solutions and cells migrating through the PLG filters showed CD44 expression consistent with that of cells before filtration. Therefore, the PLG/PVI and PLG/PLL filters are more suitable for the isolation of CI/CS cells from the perspective of CD44 marker expression experiments.

We also evaluated CD133 expression in each cell fraction after filtration through unmodified PLG filters and modified PLG (PLG/PVI and PLG/PLL) filters using colon cancer HT-29 cells (Figure 3B) and primary colon cancer PAT-3 cells (Figure 3D). The cells in the filtration solution through PLG/PVI filters showed greater CD133 expression than the cells before filtration for both HT-29 cells (Figure 3B) and PAT-3 cells (Figure 3D). The cells in the recovering solution through PLG filters showed greater CD133 expression than the cells before filtration for both HT-29 cells (Figure 3B) and PAT-3 cells (Figure 3D). The cells migrating through PLG/PLL filters showed greater CD133 expression than the cells before filtration for both HT-29 cells (Figure 3B) and PAT-3 cells (Figure 3D).

Considering that the expression of CD44 and CD133 varies in the cell fraction before and after filtration the cells migrating through the PLG/PLL filters and not the PLG filters displayed greater expression of CD44 and CD133 than the cells before filtration for both HT-29 cells (Figure 3A and B) and PAT-3 cells (Figure 3C and D).

### Immunofluorescence analysis of CI/CS cell marker expression in colon cancer cells before and after filtration

**Figure 4:**
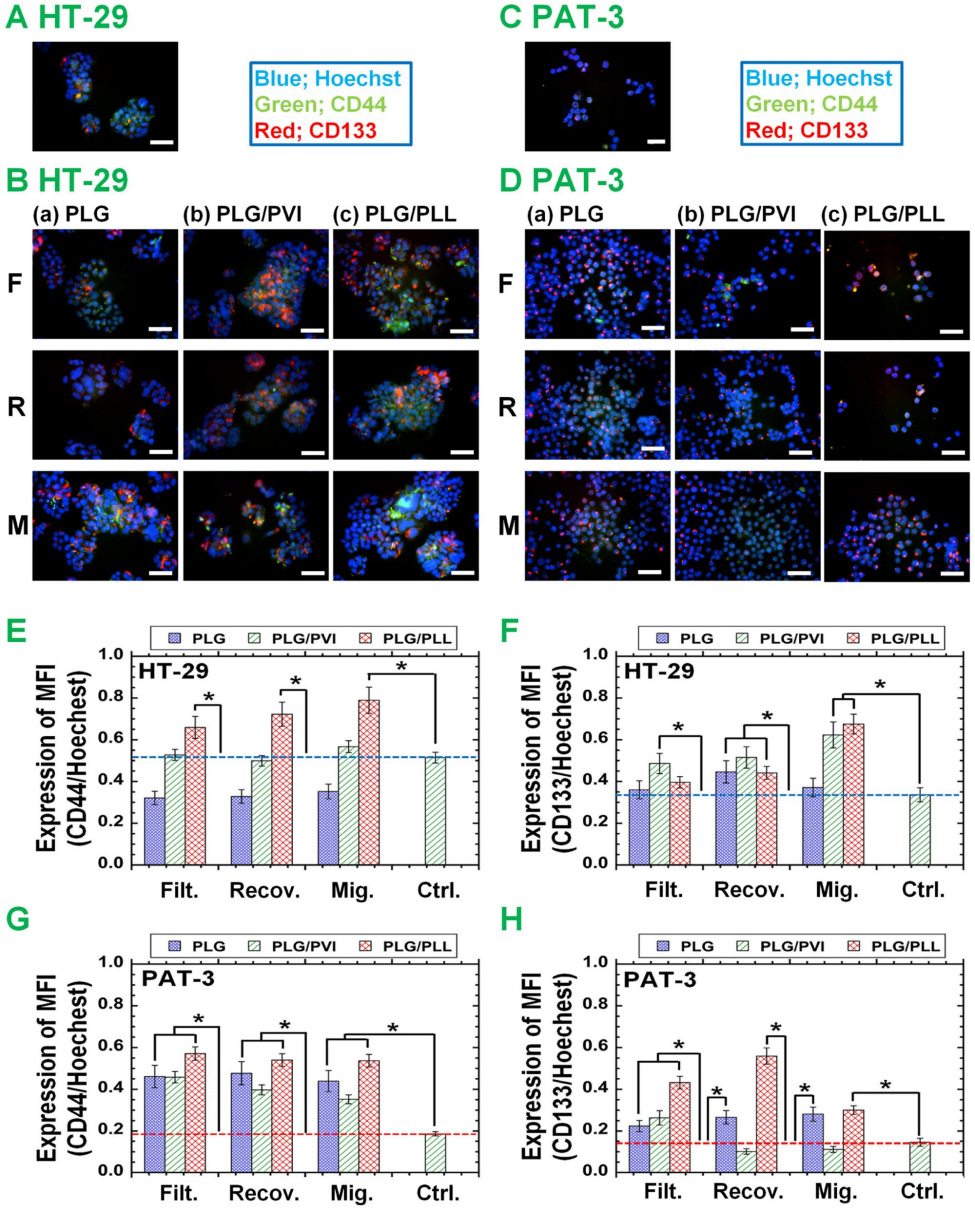
Expression of CI/CS cell markers on HT-29 and PAT-3 cells evaluated by immunostaining after filtration via unmodified and modified PLG filters (membranes). (**A**) Immunofluorescence image of the expression of the CI/CS cell markers CD44 and CD133 in colon cancer HT-29 cells on TCP dishes before filtration. (**B**) Immunofluorescence images of CI/CS cell markers CD44 and CD133 in colon cancer HT-29 cells in filtration (F) and recovering solution (R) and on migration (M) after filtration through PLG (a), PLG/PVI (b) and PLG/PLL filters (c). The scale bar indicates 100 mm. (**C**) Immunofluorescence image of CI/CS cell markers CD44 and CD133 in unfiltered PAT-3 cells on TCP dishes. (**D**) Immunofluorescence images of CI/CS cell markers CD44 and CD133 in PAT-3 cells in filtration (F) and recovering solution (R) and on migration (M) after filtration via PLG (a), PLG/PVI (b) and PLG/PLL filters (c). the scale bar indicates 100 µm. MFI (mean fluorescence intensity) of (**E**) CD44 and (**F**) CD133 relative to that of Hoechst by HT-29 cells in the filtration solution (Filt.) and recovering solution (Recov.) and on migration (Mig.) after filtration through PLG, PLG/PVI and PLG/PLL filters. MFI of (**G**) CD44 and (**H**) CD133 relative to that of Hoechst by PAT-3 cells in the filtration solution and recovering solution and on migration after through PLG, PLG/PVI and PLG/PLL filters. The dotted line shows (**E**, **G**) CD44 and (**F**, **H**) CD133 expression by unfiltered cells cultivated on TCP dishes. *P < 0.05.

We also evaluated the expression of CI/CS cell markers (CD44 and CD133) in colon cancer cells (HT-29 cells and primary PAT-3 cells) before and after filtration using immunostaining for visual (direct) observation under fluorescence microscopy. The results are displayed in Figure 4A and B for HT-29 cells and in Figure 4C and D for PAT-3 cells. The expression of each CI/CS cell marker and Hoechst (nuclear marker) was evaluated as the mean fluorescence intensity (MFI) using ImageJ, and the ratio of the MFI of CI/CS cell markers (CD44 or CD133) to that of Hoechst was calculated; the results are summarized in Figure 4E–H.

The MFI of CD44, as well as CD133, relative to that of Hoechst measured using immunostaining was very similar to the expression of CD44 and CD133 determined by FS, respectively. In particular, compared with the cells before filtration, the cells migrating through the PLG/PLL filters and not the PLG filters displayed greater MFI values for CD44 and CD133 relative to that of Hoechst for both HT-29 cells (Figure 4E and F) and PAT-3 cells (Figure 4G and H).

**Figure 5:**
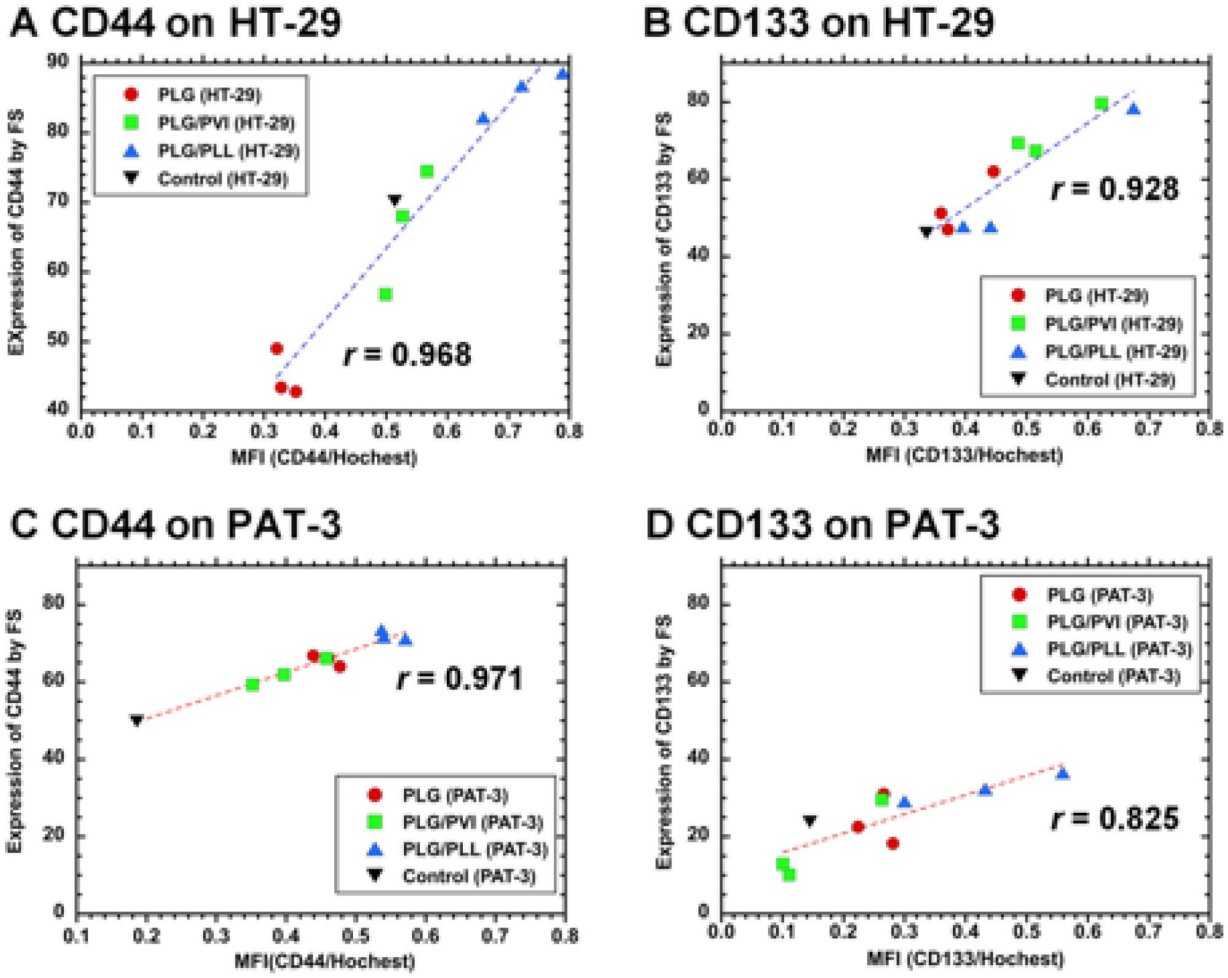
Correlation of CI/CS cell marker expression analyzed by FS and the MFI analyzed by immunofluorescence microscopy in HT-29 and PAT-3 cells. HT-29 cells were cultured on TCP dishes and analyzed for (**A**) CD44 and (**B**) CD133 expression using FS and immunostaining methods (inverted triangle); HT-29 cells in filtration and recovering solution and on migration after filtration through PLG filters (closed circles), PLG/PVI filters (closed squares) and PLG/PLL filters (closed triangles). PAT-3 cells were cultured on TCP dishes and analyzed for (**C**) CD44 and (**D**) CD133 expression using FS and immunostaining methods (inverted triangle); PAT-3 cells in filtration solution and recovering solution and on migration after filtration through PLG filters (closed circles), PLG/PVI filters (closed squares) and PLG/PLL filters (closed triangles).

Because of the similarity of CI/CS cell marker expression on the cells before and after permeation through unmodified PLG and modified PLG filters, as evaluated by FS and immunostaining, we investigated the correlation between CI/CS cell marker expression on the cells analyzed using FS and immunostaining. The results are displayed in Figure 5A for CD44 expression on HT-29 cells, Figure 5B for CD133 expression on HT-29 cells, Figure 5C for CD44 expression on PAT-3 cells and Figure 5D for CD133 expression on PAT-3 cells.

The correlation coefficient of CD44, which was analyzed using FS and fluorescence microscopy to obtain the MFI, was 0.968 for HT-29 cells and 0.971 for PAT-3 cells. Furthermore, the correlation coefficient of CD133, which was analyzed using FS and immunostaining to obtain the MFI, was 0.928 for HT-29 cells and 0.825 for PAT-3 cells. Therefore, the expression of both CD44 and CD133 in each type of colon cancer cell, namely, HT-29 and PAT-3 cells, was strongly correlated, as evaluated by FS and fluorescence microscopy (immunostaining). The MFI of specific markers on cells might be useful for researchers who are unable to use FS but can visually analyze the expression levels of specific markers using fluorescence microscopy.

### Colony formation assay of colon cancer cells before and after filtration

**Figure 6:**
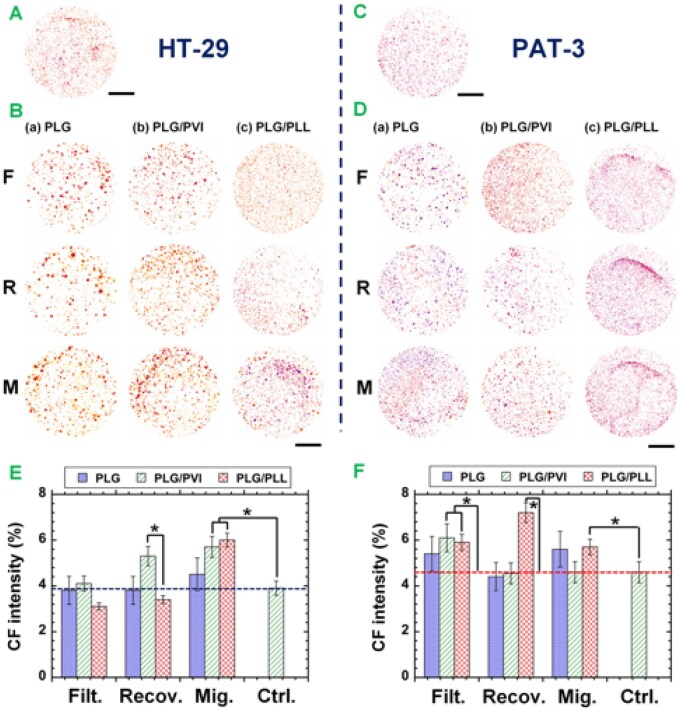
Colony formation assay of HT-29 cells and PAT-3 cells after filtration through unmodified and modified PLG filters. (**A**) Images of colonies formed by unfiltered HT-29 cells cultivated on TCP plates. The bar represents 1 cm. (**B**) Images of colonies formed by HT-29 cells in filtration solution (F) and recovering solution (R) and on migration (M) after filtration through PLG filters (a), PLG/PVI filters (b) and PLG/PLL filters (c). the bar represents 1 cm. (**C**) Images of unfiltered PAT-3 cells cultured on TCP dishes. The bar represents 1 cm. (**D**) Images of colonies formed by PAT-3 cells in filtration solution (F) and recovering solution (R) and on migration (M) after filtration through PLG filters (a), PLG/PVI filters (b) and PLG/PLL filters (c). the bar represents 1 cm. Colony-forming intensity, analyzed by ImageJ, of (**E**) HT-29 cells and (**F**) PAT-3 cells in filtration solution (Filt.) and recovering solution (Recov.) and on migration (Mig.) after filtration through PLG (left bar), PLG/PVI (middle bar) and PLG/PLL (right bar) filters. *P < 0.05.

The high colony-forming potential of cancer cells is strongly correlated with cell tumorigenicity [28, 54, 56, 60]. Therefore, we performed a colony formation assay of HT-29 cells and PAT-3 cells before and after filtration through unmodified PLG and modified PLG (PLG/PVI and PLG/PLL) filters. Images of the formation of colonies on soft agar gels by HT-29 cells (Figure 6A and B) and primary colon cancer PAT-3 cells (Figure 6C and D) in the filtration solution (Figure 6B and D) and recovering solution (Figure 6B and D) as well as migrating cells (Figure 6B and D) after filtration through the PLG, PLG/PVI and PLG/PLL filters are shown in Figure 6B and D. An image of the colony formation ability of HT-29 cells and PAT-3 cells cultivated on TCP dishes (control) is also displayed in Figure 6A for HT-29 cells and Figure 6C for PAT-3 cells. ImageJ (https://imagej.nih.gov/ij/) was used to count the number of colonies in each case, and the results are summarized in Figure 6E and F.

Similar to the expression of CD44 and CD133 on the cells, the cells migrating through the PLG/PLL filters showed greater colony-forming abilities than cells before filtration for both HT-29 cells (Figure 6E) and PAT-3 cells (Figure 6F).

**Figure 7:**
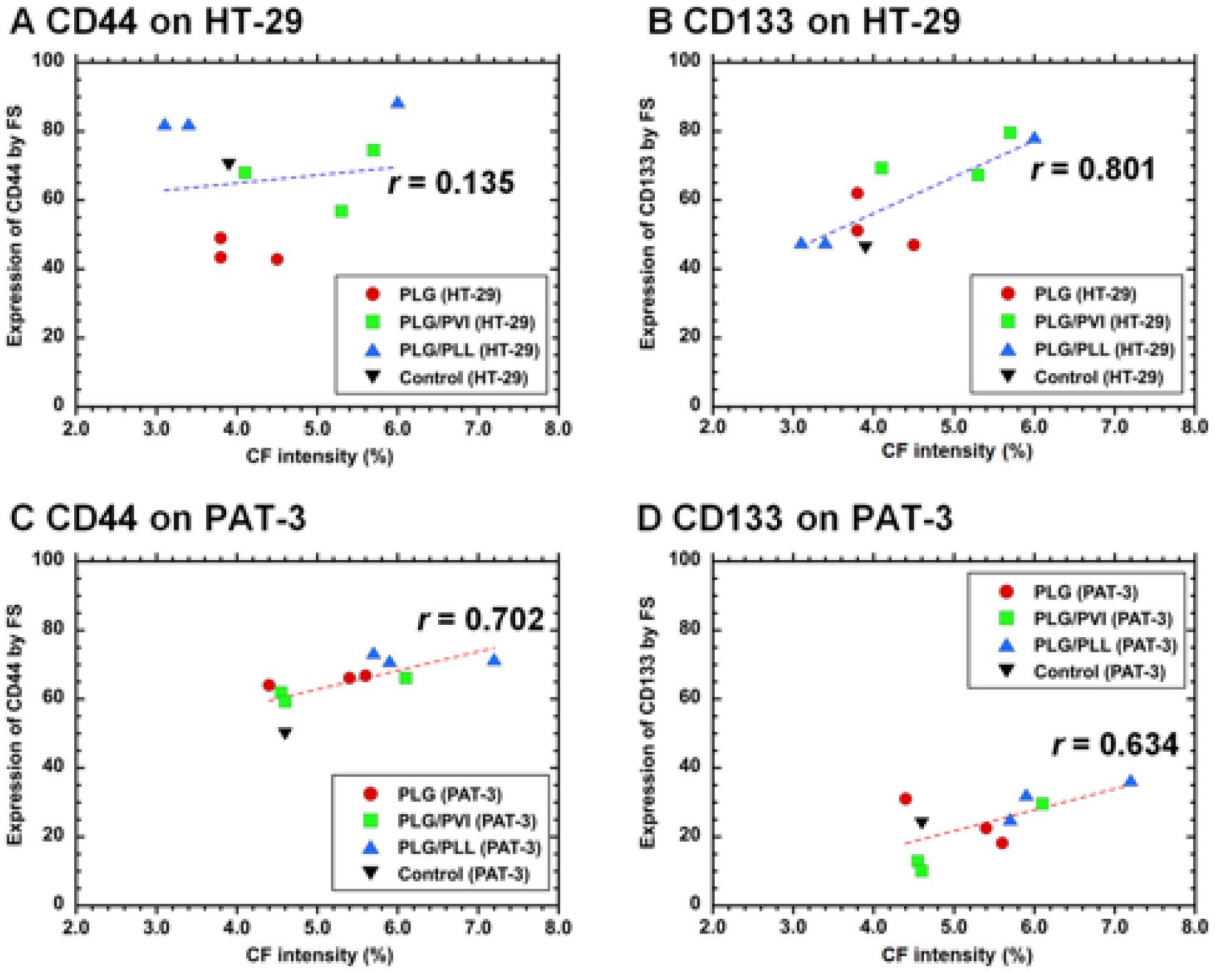
Correlation of CI/CS cell marker expression analyzed utilizing FS and the colony formation intensity determined by the colony formation assay in HT-29 cells and PAT-3 cells. HT-29 cells cultured on TCP plates (inverted triangles), in filtration and recovering solutions and on migration were analyzed for (**A**) CD44 and (**B**) CD133 expression using FS and colony formation assays, after filtration through PLG filters (closed circles), PLG/PVI filters (closed squares) and PLG/PLL filters (closed triangles). PAT-3 cells cultured on TCP dishes (inverted triangles), in filtration and recovering solutions and on migration were analyzed for (**C**) CD44 and (**D**) CD133 expression using FS and colony formation assays after filtration through PLG filters, PLG/PVI filters and PLG/PLL filters.

Thus, we investigated whether cells with high colony-forming ability express high levels of CI/CS cell markers (CD44 and CD133) before and after filtration through unmodified and modified PLG filters; the results are described in Figure 7 for HT-29 cells (Figure 7A and B) and PAT-3 cells (Figure 7C and D). The correlation coefficient between the expression intensity of CD44 and the number of colonies formed by HT-29 cells was 0.135 (Figure 7A), indicating almost no correlation. The correlation coefficient between the expression intensity of CD44 and the number of colonies formed by PAT-3 cells was 0.702 (Figure 7C), indicating a weak correlation. With respect to CD133 expression, the correlation coefficient between the expression intensity of CD133 and the number of colonies formed by HT-29 cells was 0.801 (Figure 7B), indicating a weak correlation. The correlation coefficient between the expression intensity of CD133 and the number of colonies formed by PAT-3 cells was 0.634 (Figure 7D), indicating a weak correlation. In summary, compared with CD44 expression, CD133 expression correlated more strongly with the number of colonies formed by the cells investigated in this study. This is because CD133 is more likely CI/CS cell marker of colon cancer cells than CD44 as several researchers reported [6, 16–21]. Currently, it is unknown why the migrated cells from PLG/PLL filters and not from PLG or PLG/PVI filters could contain CI/CS cells extensively. We would like to evaluate zeta potential of the migrated cells from PLL/PLG cells, which are compared with that of the cells in the filtration solution and recovering cells as well as the migrated cells from PLG and PLG/PVI filters in the future.

### CEA secretion by colon cancer cells before and after filtration

**Figure 8:**
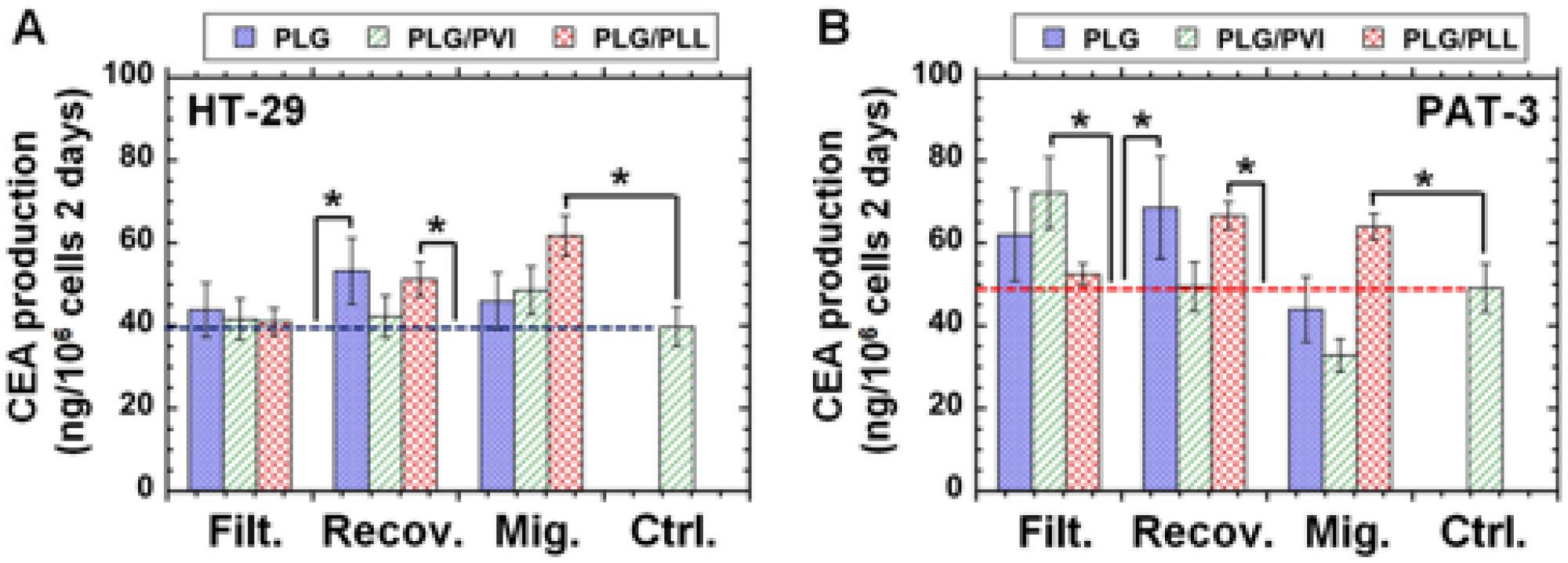
CEA production by (**A**) HT-29 cells and(**B**) PAT-3 cells on TCP plates (Ctrl; control), by cells in filtration solution (Filt.) and recovering solution (Recov.) and by migrating cells (Mig.) after filtration through PLG filters (left bar), PLG/PVI filters (middle bar) and PLG/PLL filters (right bar). the CEA production rate was evaluated after 7 days of colon cancer cell cultivation under each condition. *P < 0.05.

The secretion of CEA is among the properties of colon cancer cells. In our previous work [41, 61], cells expressing higher levels of CI/CS cell surface markers tended to secrete more CEA. Therefore, we evaluated the production of CEA by HT-29 cells and PAT-3 cells before and after filtration through unmodified PLG and modified PLG (PLG/PVI and PLG/PLL) filters, and the results are shown in Figure 8. Both HT-29 and PAT-3 cells migrating through PLG/PLL filters showed greater production of CEA than the cells before filtration. PAT-3 cells migrating through unmodified PLG filters and more negatively charged PLG/PVI filters did not show greater production of CEA than the cells before filtration.

It should be noted that CEA is considered to facilitate metastasis and block cell differentiation. Yan et al. found colon cancer cells expressing low expression of CEA showed poor prognosis and generate more tumor [62]. Jansen evaluated CEA expression in human tumors from more than 15 000 samples [63]. They found that reduced CEA expression was related to colon cancer aggressiveness. On the other hand, aggressive cancers of the urinary bladder and breast cancers showed overexpression of CEA [63]. Therefore, it should be difficult to make the correlation between the colon cancer cells containing high CI/CS cells and CEA production of the colon cancer cells in this study. In contrast, we proceeded with a xenograft tumorigenicity evaluation by subcutaneous transplantation of colon cancer cells filtered through the filters in the following experiments.

### A xenograft tumorigenicity after the transplantation of filtered primary cancer cells into mice

After filtration through positively charged PLG/PLL filters, both HT-29 and PAT-3 cells presented greater CD44 and CD133 expression (Figures 3 and 4) and colony-forming ability (Figure 6) than after filtration through unmodified PLG filters and more negatively charged PLG/PVI filters. Therefore, we used PAT-3 cells filtered through positively charged PLG/PLL membranes for the *in vivo* evaluation of xenograft tumorigenicity, which is a direct method for quantifying colon CI/CS cells *in vivo*. This is because PAT-3 is primary colon cancer cells isolated from colon cancer patients in this study. Our final goal is to investigate whether colon cancer cells of patients are high or low malignant (contain high or low CI/CS) cells from evaluation of patient tumor tissues and also to evaluate which drugs are effective in killing CI/CS cells in specific patient cancer cells (personalized drug treatment). Therefore, we evaluated PAT-3 cells *in vivo*, of which results are more impactful than conventional colon cancer cell line of HT-29 cells to save usage of mice and research funding.

**Figure 9:**
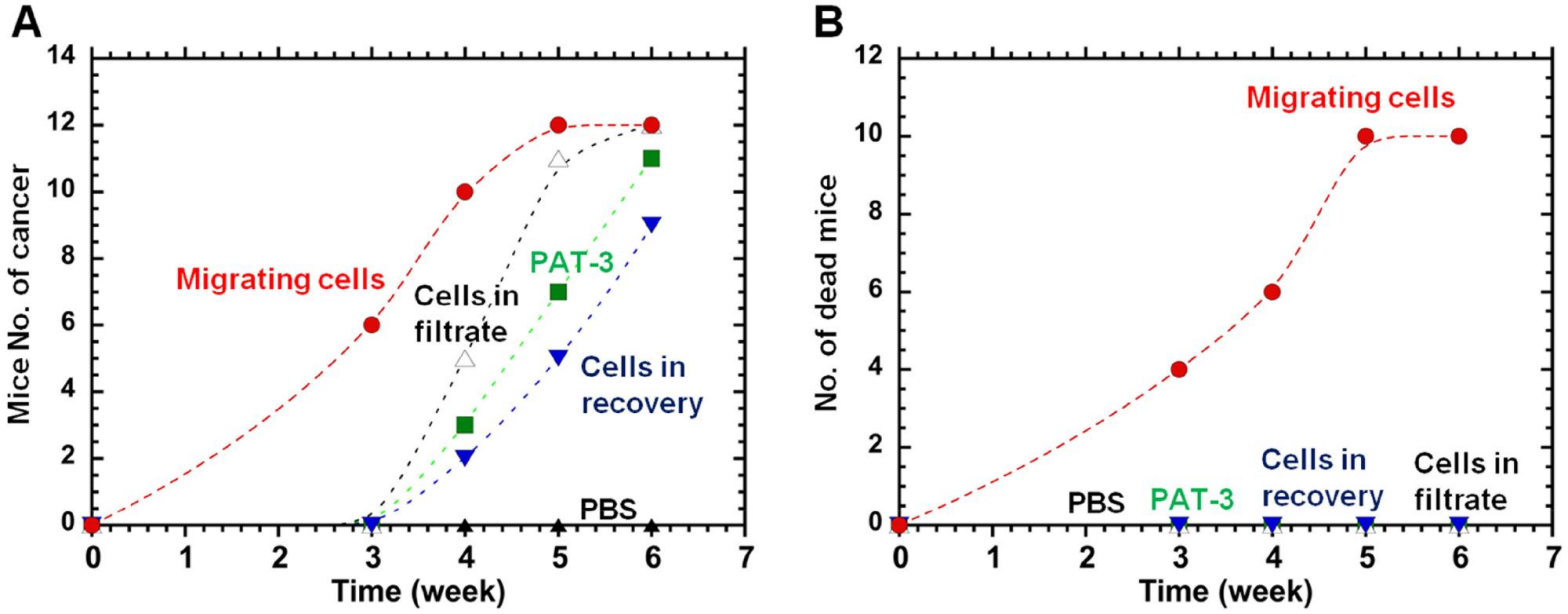
A xenograft tumorigenicity by injection of PAT-3 cells before and after filtration. (**A**) No. of tumor-generating mice after the subcutaneous injection of PBS (closed triangles; negative control), unfiltered PAT-3 cells (closed squares), PAT-3 cells in filtration solution (open triangles), PAT-3 cells in recovering solution (inverted closed triangles) and migrating PAT-3 cells (closed circles). (**B**) No. of dead mice after the subcutaneous injection of PBS (closed triangles; negative control), unfiltered PAT-3 cells (closed squares), PAT-3 cells in filtration solution (open triangles), PAT-3 cells in recovering solution (inverted closed triangles) and migrating PAT-3 cells (closed circles).

PAT-3 cells (8.0 × 10^6^) filtered through PLG/PLL filters were injected subcutaneously into nonobese diabetic/severe combined immunodeficiency (NOD-SCID, NOD.CB17-Prkdcscid/NcrCrl) mice, and the results are shown in Figure 9 and Supplementary Figure S1. Twelve mice were evaluated under each condition: injection with (a) PBS (negative control); (b) unfiltered PAT-3 cells; (c) PAT-3 cells in filtration solution; (d) PAT-3 cells in recovering solution; or (e) migrating PAT-3 cells.

Mice subcutaneously injected with PBS (negative control) did not generate tumors; in contrast, all mice injected with migrating PAT-3 cells filtered through PLG/PLL generated tumors and generated them faster (Figure 9A). In particular, 3 weeks after subcutaneous cell injection, only the mice injected with migrating cells generated tumors, whereas no tumors were generated in the mice under the other conditions. PAT-3 cells in recovering solution generated tumor cells at a slightly slower speed than unfiltered PAT-3 cells did, whereas PAT-3 cells in filtration solution generated tumors slightly faster than unfiltered PAT-3 cells.

We also evaluated the cancer-related mortality rate, and the results are shown in Figure 9B. Only mice injected with cells migrating through PLG/PLL died during the experiments [10 of 12 mice (83%) died 6 weeks after the injection of migrating PAT-3 cells], whereas all other mice, including those injected with PAT-3 cells in filtrate and recovering solutions, unfiltered cells and PBS, survived. Therefore, we concluded that the migrating PAT-3 cells through PLG/PLL are the most malignant and contain more CI/CS cells.

Tumor volume enhancement of the mice after injection of filtered and unfiltered PAT-3 cells was also investigated in this study and is shown in Supplementary Figure S2. Tumor volume of the mice injected with the PAT-3 migrated cells was the highest among those injected with filtered or unfiltered PAT-3 cells at 5 weeks post injection (*P *< 0.00001) (Supplementary Figure S2). However, the tumor volume of the mice injected with the migrated cells was almost the same compared to that injected with unfiltered PAT-3 cells at 6 weeks post injection (*P *> 0.999) (Supplementary Figure S2). This is because 8 mice among 12 mice were already dead, which were injected with the PAT-3 migrated cells and the dead mice tumor volume was not counted in this figure (Supplementary Figure S2), whereas no dead mice among 12 mice was found at 6 weeks of post injection, which were injected with unfiltered PAT-3 cells (Figure 9B).

As the limitation of this research, we only used one primary colon cancer cell line for tumor formation after the transplantation of filtered primary colon cancer cells into mice in this study. This is because it is difficult to establish primary colon cancer cell lines from patient colon cancer tissue. We will evaluate tumor formation after the transplantation of filtered primary colon cancer cells into mice using different primary colon cancer cells established from different patients in the future.

## Conclusion

We designed positively charged PLG/PLL filters and negatively charged PLG/PVI filters for the purification of CI/CS cells from colon cancer cells. Colon cancer cells, including commercially available HT-29 colon cancer cells and PAT-3 primary colon cancer cells, that migrated through PLG/PLL filters presented greater expression of the colon cancer CI/CS cell markers CD44 and CD133, greater colony-forming ability, greater CEA production *in vitro* and greater xenograft tumorigenicity *in vivo* than colon cancer cells without filtration. Positively charged PLG/PLL filters efficiently enriched CI/CS cells from colon cancer cells. The PLG/PLL filtration method is very effective for purifying CI/CS cells from colon cancer cells. PLG was already approved for use as a biocompatible and biodegradable polymer from The US Food and Drug Administration (FDA) and FDA conferred similar materials to ε-polylysine (PLL), as GRAS, “Generally Recognized as Safe” status to be used into various food items at levels of 10–500 ppm [64], which indicates PLL is a relatively safe material. Therefore, it should be not so difficult that PLG/PLL filters are approved by FDA [65] as a medical device for CI/CS isolation from patient’s colon cancer tissue solution in the future, although reproducibility and uniform production of PLG/PLL filters should be investigated as a medical product. RMSR value of PLG/PLL (65.9 ± 8.8 µm) is similar to that of PLG/PVI (76.7 ± 26.6 µm), which is shown in Table 1. Therefore, the isolation efficiency of CI/CS cells from colon cancer cells seems not to depend on the RMSR value of the filters in this study. We remain to investigate the effect of pore size of PLG/PLL filters on the isolation of CI/CS cells. However, this study showed the membrane filtration method combined with charge-based cell sorting is effective to isolate CI/CS cells (CD44^+^CD133^+^ cells) from colon cancer cells. In the future, CI/CS cells could be isolated more efficiently by the optimization of the pore size of PLG/PLL filters. We are now starting to isolate CI/CS cells from ocular cancer tissues, such as ocular melanoma, retinoblastoma, conjunctival squamous cell carcinoma and/or medulloepithelioma tissues, using the developed filtration method.

## Supplementary Material

rbag018_Supplementary_Data

## Data Availability

Data will be made available up request.
