# Supplementary material for: Enrichment of colon cancer stem cells via polymeric porous filters with different zeta potentials
Source: Regen Biomater. 2026 Feb 9;13:rbag018. doi: 10.1093/rb/rbag018 (PMC13012879; doi:10.1093/rb/rbag018)
Supplement: rbag018_Supplementary_Data [file rbag018_supplementary_data.pdf]

Enrichment of colon cancer stem cells via polymeric porous filters with different zeta potentials

Tzu-Cheng Sung[1,†], Ling-Chun Hung[2,†], Min Gao[1], Xuanyu Lin[1], Manman Yang[1], Xin Kang[1], Zeyu Tian[1], Ting Wang[1], Jian Gong[3], Jiandong Pan[1], Henry Hsin-Chung Lee[4,5] Akon Higuchi[1,2,6,*]

[1]State Key Laboratory of Eye Health, Eye Hospital, Wenzhou Medical University, No. 270, Xueyuan Road, Wenzhou, Zhejiang, 325027, China

[2]Department of Chemical and Materials Engineering, National Central University, No. 300, Jhongda RD., Jhongli, Taoyuan 32001, Taiwan, China

[3]Department of Clinical Laboratory, The Second Affiliated Hospital and Yuying Children's Hospital of Wenzhou Medical University, No. 109, Xueyuan West Road, Lucheng, Wenzhou, Zhejiang, 325027, China

[4]Department of Surgery, Cathay General Hospital, 280 Jen Ai Road, Section 4, Taipei, 106, Taiwan, China.

[5]Graduate Institute of Translational and Interdisciplinary Medicine, National Central University, No. 300, Jhongda Rd., Jhongli, Taoyuan, 32001, Taiwan, China.

[6]R&D Center for Membrane Technology, Chung Yuan Christian University, Chungli, Taoyuan 320, Taiwan, China

* Corresponding author. State Key Laboratory of Eye Health, Eye Hospital, Wenzhou Medical University, No. 270, Xueyuan Road, Wenzhou, Zhejiang, 325027, China

Tel.: +86 577-88068822; fax: +86 086-577-88832083.

E-mail address: higuchi@ncu.edu.tw; higuchi@wmu.edu.cn (A. Higuchi)

[†] These authors contributed equally.

Supplementary Information

Table S1 Materials used in this study.

Materials	Abbreviation	Catalog No.	Company
		**Polymer**	
Poly(lactide-co-glycolic acid) (lactide:glycolic=75:25)	PLG	P1941	Sigma-Aldrich (St. Louis, MO, USA)
Poly(vinyl alcohol-co-itaconic acid)	PVI	PVA-IA	Japan Vam & Poval Co. (Osaka, Japan)
Poly-L-lysine	PLL	P7890	Sigma-Aldrich (St. Louis, MO, USA)
Silk screen mesh	Silk screen mesh (Teflon)	180 mesh	Yuzawaya, Tokyo, Japan
2-Hydroxyethyl agarose	Agarose	39346-81-1	Sigma-Aldrich (St. Louis, MO, USA)
		**Animal & cell**	
NOD.CB17-Prkdcscid/NcrCrl mice	NOD-SCID mice	406	Charles River Laboratories (Beijing, China)
HT-29	HT-29	60157	BCRC, Food Industry Research and Development Institute (Hsinchu, Taiwan, China)
		**Cell culture dishes**	
6-well tissue culture polystyrene plate	TCP	353046	Corning (Corning, NY, USA)
Polystyrene dish	TCP	430165	Corning (Corning, NY, USA)
Antibiotic-antimycotic	anti-anti	15240096	Thermo Fisher Scientific Inc. (Waltham, MA, USA)
		**Chemicals**	
High-vacuum grease	High-vacuum grease	1658832	Dow Corning Corporation (Midland, MI, USA)
Human CREA ELISA kit	Human CREA ELISA kit	EHCEA	Thermo Fisher Scientific Inc. (Waltham, MA, USA)
		**Cell culture medium and component**	
DMEM	DMEM	D5648-10x1L	Sigma-Aldrich (St. Louis, MO, USA)
Fetal bovine serum	FBS	04-001-1A	Biological Industries, Kibbutz Beit-Haemek, Israel
Hoechst 33342	Hoechst	PA-3014	Lonza (Basel, Switzerland)
		**Surface markers**	
7-AAD Viability dye	7-AAD	559925	BD Biosciences (San Jose, CA, USA)
FITC mouse anti-human CD44	FITC anti-CD44	555478	BD Biosciences (San Jose, CA, USA)
PE mouse anti-human CD133/1	PE anti-CD133	130-080-801	Miltenyi Biotech (Bergisch Gladbach, North Rhine-Westphalia, Germany)
FITC Mouse IgG2bκ, isotype control	FITC isotype	555742	BD Biosciences (San Jose, CA, USA)
PE Mouse IgG1κ, isotype control	PE isotype	555749	BD Biosciences (San Jose, CA, USA)

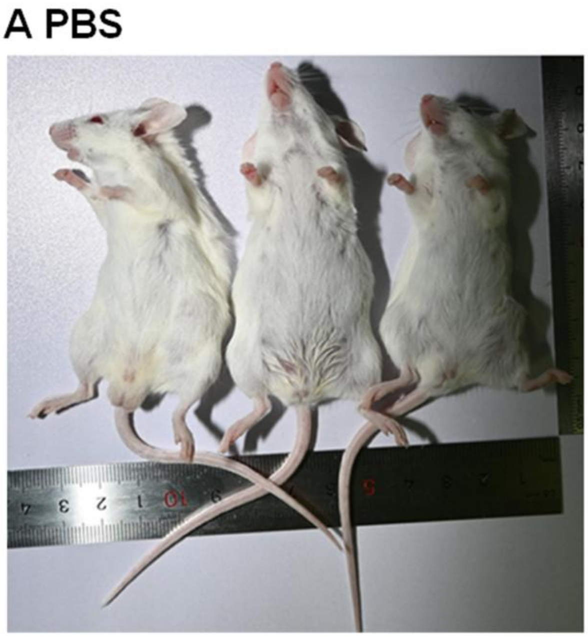

Supplementary Fig. 1 Representative images of tumor generation in mice three weeks after the subcutaneous injection of PBS (i) and cells migrating from PLG/PLL filters (ii). The arrow indicates the tumor.

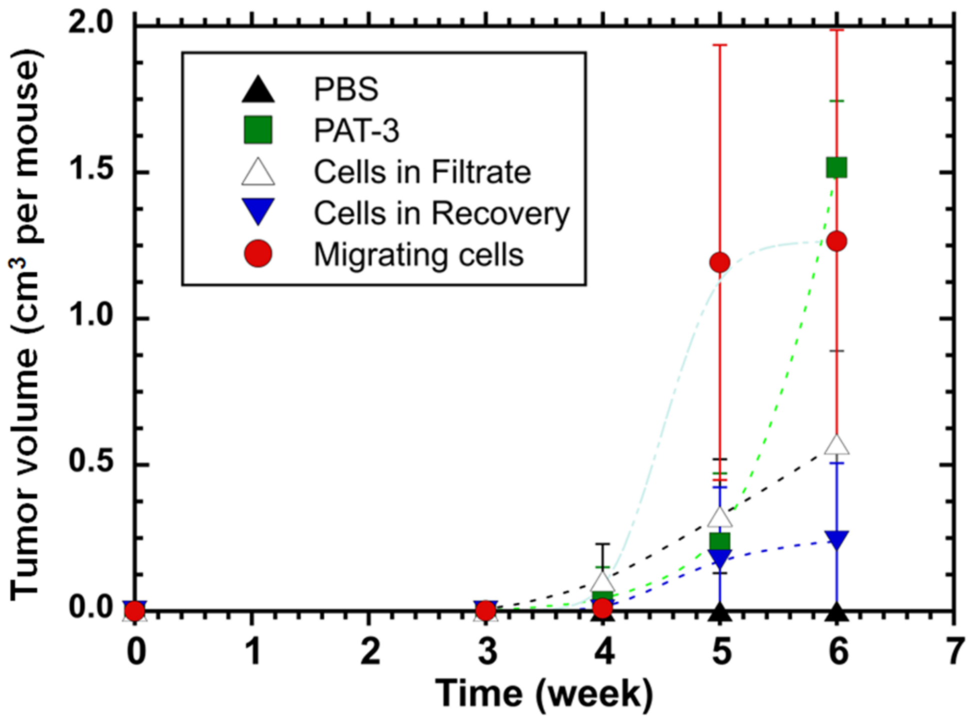

Supplementary Fig. 2 Tumor formation by injection of PAT-3 cells before and after filtration. Tumor volume of mice after the subretinal injection of PBS (closed triangles) (negative control), unfiltered PAT-3 cells (closed squares), PAT-3 cells in filtration solution (open triangles), PAT-3 cells in recovering solution (inverted closed triangles), and migrating PAT-3 cells (closed circles).
